# SCUBA Diving in Adult Congenital Heart Disease

**DOI:** 10.3390/jcdd10010020

**Published:** 2023-01-04

**Authors:** Robert M. Kauling, Rienk Rienks, Judith A. A. E. Cuypers, Harald T. Jorstad, Jolien W. Roos-Hesselink

**Affiliations:** 1 Department of Cardiology, Thorax Center, Erasmus MC, University Medical Center Rotterdam, 3015 GD Rotterdam, The Netherlands; 2 CardioExpert Clinic for Sports and Occupational Cardiology, 1087 DK Amsterdam, The Netherlands; 3 Department of Cardiology, University Medical Center Utrecht, 3584 CX Utrecht, The Netherlands; 4 Department of Cardiology, Heart Center, Amsterdam UMC Location AMC, University of Amsterdam, Amsterdam Movement Sciences, Amsterdam Cardiovascular Sciences, 1105 AZ Amsterdam, The Netherlands; 5 European Reference Network for Rare, Low Prevalence and Complex Diseases of the Heart (ERN GUARD-Heart), 1105 AZ Amsterdam, The Netherlands

**Keywords:** scuba diving, congenital heart disease, fitness to dive

## Abstract

Conventionally, scuba diving has been discouraged for adult patients with congenital heart disease (ACHD). This restrictive sports advice is based on expert opinion in the absence of high-quality diving-specific studies. However, as survival and quality of life in congenital heart disease (CHD) patients have dramatically improved in the last decades, a critical appraisal whether such restrictive sports advice is still applicable is warranted. In this review, the cardiovascular effects of diving are described and a framework for the work-up for ACHD patients wishing to engage in scuba diving is provided. In addition, diving recommendations for specific CHD diagnostic groups are proposed.

## 1. Introduction

Scuba diving is an increasingly popular activity. The Professional Association of Diving instructors (PADI) which covers 60–70% of the global scuba diving market, has issued more than 28 million diver certifications globally since 1976 [1]. Because of this increased popularity, there has also been an increase in people with cardiovascular disease who wish to start diving, including patients with (repaired) congenital heart disease (CHD). This topic has become more relevant as the number of adults with CHD (ACHD) is expanding, with more than 90% of children born with CHD reaching adulthood nowadays [2,3,4]. Most of these patients experience a good quality of life, and want to live a normal life, including sports participation, and a growing number of ACHD patients consider scuba diving [5,6]. As such, cardiologists are frequently confronted with ACHD patients seeking advice whether they are fit to dive. 

Recently, the Task Force on Sports Cardiology and exercise in patients with cardiovascular disease of the European Society of Cardiology (ESC) published updated guidelines regarding the participation in competitive sports of patients with cardiovascular disease [7]. Although some general contra-indications for diving are included in this guideline, eligibility for scuba diving is only mentioned briefly. More specific recommendations regarding participation in competitive sports in ACHD patients were recently published in a position paper by the Working Group on Adult Congenital Heart Disease and on Sports Cardiology [8]. In this document, scuba diving has been classified as a “skill sports discipline”, due to its presumed limited effects on heart rate and blood pressure, but with an intrinsic risk of serious harm or death for the athlete in the event of syncope. 

The classification of scuba diving as a skill sport has profound implications for the evaluation of fitness-to-dive in patients with ACHD. The spectrum of ACHD and its associated complications range from conditions conventionally considered as incompatible with scuba diving (e.g., pulmonary hypertension, cyanosis and ventricular hypertrophy) to other conditions which may be compatible with recreational scuba diving. Severity of ACHD differs substantially amongst and within different diagnosis groups. As such, current recommendations may seem arbitrary and lack room for individual considerations. There is currently no ACHD-specific framework on how to assess this patient group specifically for fitness-to-dive, and practical recommendations as to the ACHD patient and diving are lacking. In this review, we therefore discuss the general physiology of diving, provide tools for the work-up and evaluation of fitness to dive in patients with ACHD and provide decisive elements to advise for or against starting this activity. 

## 2. Diving Physiology: Cardiovascular and General Aspects

Immersion and submersion have profound effects on the cardiovascular, respiratory, endocrinal, renal and central nervous systems. In healthy individuals, immersion leads to an increase in hydrostatic pressure, resulting in a fluid-shift towards the central circulation, consequently increasing the preload of the left ventricle augmenting cardiac output via the Frank–Starling mechanism. This volume loading of the ventricles leads to right atrial and ventricular dilatation, with a compensatory increase in the secretion of natriuretic peptide. Throughout the dive, this will gradually increase diuresis (“diver’s pee”), resulting in a relatively hypovolemic state at the end of the dive [9]. Secondly, even in tropical waters, immersion and submersion will induce peripheral vasoconstriction, resulting in a rise in systolic blood pressure and ventricular afterload. 

Finally, exposure of the trigeminus nerve to cold water will stimulate the diving reflex. This will induce inhibition of the cardio-respiratory center in the medulla oblongata, resulting in bradycardia (decrease in the heart rate of up to 60%), QT interval prolongation and vasoconstriction [10]. The combination of sympathetic and parasympathetic stimulation may in rare cases lead to an “autonomic conflict”, which has been associated with the onset of ventricular arrhythmias [11]. This might lead to life-threatening arrhythmias in vulnerable hearts, for instance with hypertrophy, ischemia, pre-existing arrhythmias and channelopathies [11].

In patients with cardiovascular disease, diving-associated hemodynamic alterations potentially disturb a previously well-tolerated cardiac condition. In particular in patients with diminished right or left ventricular systolic function, restrictive diastolic function or patients with moderate to severe valvular disease, these volume shifts might lead to cardiac decompensation, and pulmonary oedema may develop (immersion pulmonary oedema (IPE)) [12]. The development of a bradycardia by the diving reflex might be especially relevant in patients with a depressed systolic left or right ventricular function, resulting in a decrease in cardiac output. In addition, peripheral vasoconstriction will result in an increase in systemic afterload and may challenge divers with a left ventricular outflow tract (LVOT) obstruction or depressed left ventricular function. 

In addition to these cardiovascular effects, several general aspects are important in all divers. Water is in principle an unforgiving environment. To survive, one needs an air supply, whether from the surface (with an “umbilical”), or from a carried tank filled with air (or another gas mixture). In the water there are hyperbaric conditions. At every 10 meters of increasing depth, the ambient pressure increases by 1 atmosphere. As a result, inhalation gases become denser, increasing the respiratory work [13]. Additionally, the solubility of the gases increases. This results in storage of especially nitrogen from the air in the blood and tissues. 

The main factors for this build-up are the nitrogen content of the inhaled gas, depth of the dive and duration of the dive. One can calculate how much nitrogen is being stored in the tissues during the dive, and how long and how deep one can dive without extra stops to wash-out the nitrogen from the tissues. These calculations are available as “no decompression dive tables” (no-deco dives). When exceeding the limits as to depth and time indicated by these tables, chances of decompression sickness (DCS) increase. All patients are advised to remain within the limits of these decompression tables. 

After the dive, when ambient pressure has been reduced to normal (1 atmosphere), this nitrogen is released from the tissues. This may cause local bubble formation, resulting in local DCS (for instance in the joints, “the bends”). The nitrogen may also be transported through the blood to the lungs, where it is exhaled. When there is too much nitrogen in the blood, bubble formation may result. These bubbles cause a temporary increase in pulmonary artery pressure [14]. When there is a connection between the pulmonic and systemic circulation, for instance by means of a patent foramen ovale (PFO), these bubbles may enter the systemic circulation and cause DCS. The symptoms of shunt-related DCS are dependent on the organ involved, for instance, neurological syndromes, unconsciousness, vertigo or cutis marmorata. DCS should be differentiated from Arterial Gas Embolism (AGE), which occurs when expanding gas stretches and ruptures alveolar capillaries (pulmonary barotrauma) allowing alveolar gas to enter the arterial circulation [15].

## 3. Exercise Capacity and Diving

Diving exercise intensity was recently reported to be 5 ± 2 METs in 139 experienced recreational divers (age 42 ± 10 years, total 959 dives), leading to the suggestion that maintaining an exercise capacity of >7 METs (peak VO2 24.5 mL/kg/min in men and 22.4 mL/kg/min in women) would generally be adequate for uncomplicated recreational diving [16]. However, in some circumstances, a higher energy expenditure is required, for instance in case of a difficult entry or exit from the diving place (long walk with the equipment that may weight up to 20–30 kg), waves or currents. Of particular importance is that a diver should be able to rescue his or her buddy diver, which may require a substantially higher exercise capacity. Professional diving organizations (military, police, fire brigade) usually require an exercise capacity of 13 METs (peak VO2 40 mL/kg/min) [17]. Patients (whether with ACHD) who do not meet an exercise capacity of 25 mL/min/kg are conventionally advised not to dive, while patients with a VO2 max between 25 and 40 (men) or 25 and 35 (women) are advised to engage in non-strenuous diving or fitness optimization prior to commencing diving [18].

## 4. Evaluating Fitness to Dive in Patients with ACHD

Many patients with ACHD will be under regular follow-up by their own congenital cardiologist. Medical Examiners of Divers (MED) should obtain information from the treating physician during the evaluation process. Topics that should be discussed are the medical history, current cardiovascular status (including exercise capacity and echocardiography), medication use and any cardiovascular complications disqualifying the candidate for diving. The focus of this information should be on how diving physiology potentially may lead to a deterioration in cardiac function and thus threaten the patient or his/her diving-buddy. Patients with ACHD who are approved fit-to-dive after thorough evaluation should have an annual reassessment by their MED, to rule out any onset of new symptoms or signs of cardiac deterioration. 

Comparable to a general cardiovascular diving evaluation, a detailed history-taking and physical examination should be performed. Chest pain, dyspnea, dizziness, palpitations or loss of consciousness, especially during exercise, disqualify candidate-divers pending further evaluation. 

Many ACHD patients have undergone one or more surgical procedures involving a sternal and/or lateral thoracotomy. If the surgery involved the pleura, the risk of persisting pleural adhesions remains. Patients after thoracotomy should undergo pulmonary function testing and when abnormal should also have a CT thorax, and in case of abnormalities (for instance adhesions) be referred to a pulmonologist. Patients who have undergone a lateral thoracotomy are advised to have a CT thorax in any case, because of the perceived risk of pleural adhesions. Patients with any pleural adhesions should be advised against diving [19,20]. 

Physical examination should include at least office blood-pressure and cardiac examination, focusing on signs of heart failure and the presence of (new) cardiac murmurs. As hypertension is associated with IPE, patients with a blood pressure exceeding 160/100 mmHg at rest should be advised against diving until adequate treatment of blood pressure has been achieved [21,22]. Any signs of heart failure or the presence of previously unknown cardiac murmurs should prompt further evaluation before certification.

A recent 12-lead electrocardiogram is mandatory. New onset supraventricular arrhythmia temporally disqualifies a patient from diving pending further analysis and adequate treatment. If the ECG shows new signs of left or right ventricular hypertrophy, especially in patients known to have abnormal loading conditions, echocardiographic evaluation is indicated to rule out any progression in valvular abnormalities or the development of hypertrophic cardiomyopathy. In addition, new onset AV block or bundle branch block warrants further analysis with advanced AV block being a contra-indication for diving [23].

Specific recommendations in patients with cardiac arrhythmias are provided in the section below covering specific conditions. 

Transthoracic echocardiography is essential in the evaluation of candidate-divers with ACHD, and a recent (<12 months) echo study should be available in all patients. Basic chamber quantification with assessment of the left and right ventricular dimensions and function, aortic dimensions, valvular function and estimation of the pulmonary pressures should be performed [24,25,26]. Echocardiography of the right ventricle in ACHD patients can be challenging. The right ventricle’s unique crescent shape complicates quantification of its size and function by echocardiography. By using multiple acoustic windows, both qualitative and quantitative parameters could be examined [24]. Specific considerations with respect to echocardiographic assessment of several common forms of congenital heart disease are discussed separately in the section on specific conditions. Echocardiographic aspects and cut-offs that are important in all ACHD patients are summarized in Table 1. In this table, results compatible with diving are marked green while results that are incompatible with diving are marked red. Results marked orange are conditionally compatible with diving. 

In all patients with suspected aortic dilatation, we recommend performing CT or MR angiography to determine exact aortic dimensions and evaluate the parts of the thoracic aorta that are poorly visualized by echocardiography.

Cardiopulmonary exercise testing is recommended in all patients with ACHD, with a recommended minimum functional capacity of 8 METs to allow diving. In general, most ACHD patients report normal or only mild limitations in self-reported exercise capacity [28,29,30]. However, self-reported exercise capacity is unreliable in ACHD patients because patients are used to this situation and judge their exercise as normal, while in fact being suboptimal to poor, depending on the diagnosis [28,29,31,32,33]. In general, ACHD patients demonstrate a lower peak VO2 compared with the predicted peak VO2 for sedentary individuals of the same age and gender, ranging from 12.2 ± 3.8 mL/min/kg in patients with Eisenmenger syndrome to 31.9 ± 9.2 mL/min/kg in transposition of the great arteries (TGA) after arterial switch [33]. Interestingly, even adults with CHD in New York Heart Association (NYHA) class 1 showed an impaired exercise capacity compared with healthy subjects of a similar age (peak VO2 21.7 ± 8.5 versus 45.1 ± 8.6 mL/min/kg) [32]. General disqualifying features for diving during exercise testing, such as rhythm or conduction disorders, insufficient increase in blood pressure and ischemic changes also apply for patients with ACHD. Specific attention should be paid to an abnormal (hypertensive) blood pressure response (>250 mmHg), which disqualifies from diving. A drop in transcutaneous arterial oxygen saturations during exercise below 95% should prompt attention to previously unknown cardiac shunts or relevant pulmonary disease and disqualifies a patient for diving. 

ACHD patients with a reduced exercise capacity should realize that they are not suitable for participating in all types of diving at all places. They should be advised to avoid diving in circumstances requiring strenuous exercise, such as when there are strong currents, cold water, difficult entry into and exit from the water, carrying heavy equipment such as twinsets and decompression cylinders, stress dives, cave and wreck diving, technical diving and being an instructor for other divers [18].

**Figure 1:**
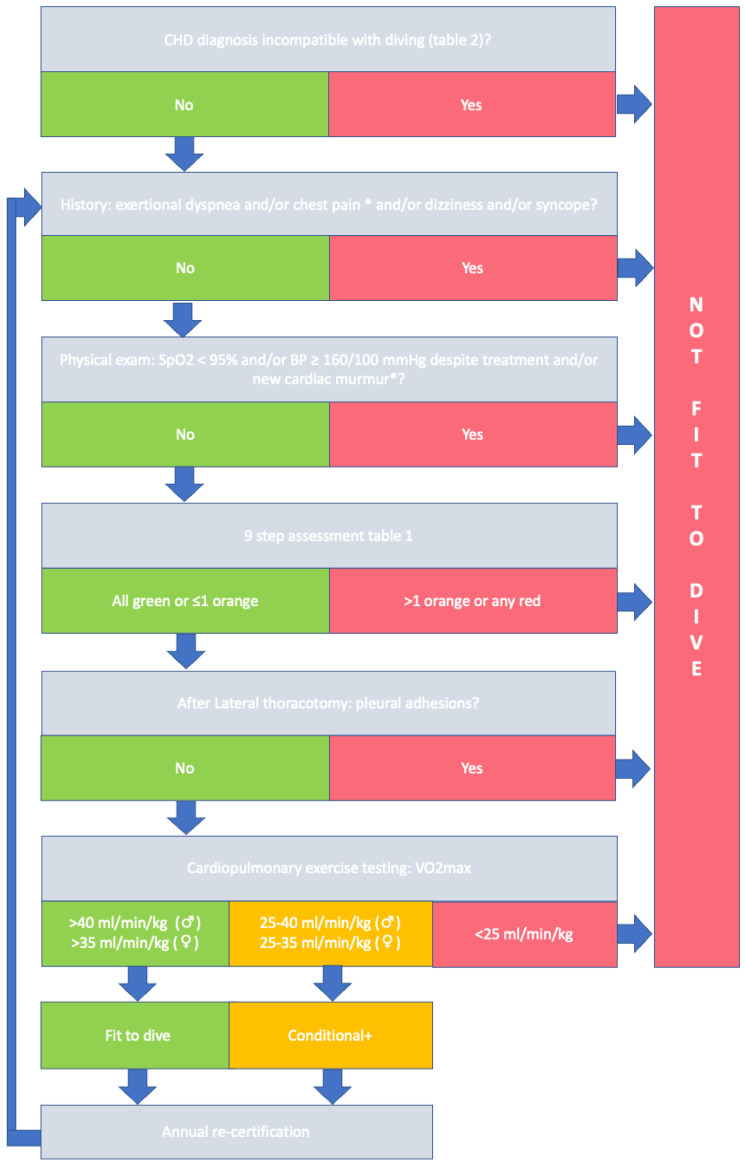
Evaluating fitness-to-dive in ACHD patients. * Reassessment after clinical work-up. + Conditional, non-strenuous diving.

In accordance with the recently published ESC guidelines on sports cardiology and recommendations of the working group of Adult Congenital Heart Disease, candidate-divers should be evaluated according to a stepwise approach [7,8]. In concordance with this approach, in Figure 1 a flow-chart is provided describing our framework for evaluation of a candidate-diver with ACHD. 

## 5. Specific Conditions 

In the upcoming section, specific CHD diagnoses in relation to diving are discussed. An overview of conditions that are incompatible with diving are provided in Table 2. 

### 5.1. Atrial Septal Defect (ASD) 

Patients with an uncorrected ASD or residual ASD should be counselled against diving because of the increased risk of right to left shunting and hence development of neurological complications due to DCS. The exact incidence of DCS in ASD patients is unknown. However, the risk of suffering a DCS is related to the size of the atrial shunt rather than just the presence of the defect itself [34]. This poses the question whether selected patients, after extensive consultation and with only a small defect, are allowed to dive. Currently there are no prospective studies evaluating diving safety in ASD patients available. It seems reasonable that conditional approval of such patients, with conservative (“low bubble diving”) could be considered, to minimize the build-up of nitrogen in the tissues (Box 1).

Box 1 **Conservative diving (low-bubble diving).**

- *Use of nitrox gas (higher oxygen, lower nitrogen content), with decompression times calculated on air tables*
- *No deep dives (>25 m)*
- *No repetitive dives*
- *Minimization of Valsalva maneuvers*
- *No decompression dives*

*or*

- *Depth of 15-meter breathing air or equivalent:*
  ○ *19 msw with nitrox 32*
  ○ *23 msw with nitrox 40*

Patients after previous successful ASD closure (surgically or percutaneously) are allowed to dive provided that residual shunting is ruled out with an echocardiographic evaluation, including bubble contrast. After recent surgical or percutaneous closure, patients should not dive for 12 and 6 months, respectively, with careful evaluation of residual shunting and cardiopulmonary status before starting diving. Although pulmonary hypertension after successful ASD closure (with normal pulmonary pressures before closure) is rare, an elevated pulmonary pressure should be ruled out [35]. In ASD patients with palpitations, there should be a low threshold for ambulant 24-h Holter monitoring since there is a high incidence of atrial arrhythmias in ASD patients during long term follow-up [30]. Cardiopulmonary exercise testing is indicated, although in general, exercise capacity in patients after ASD closure is adequate [30,36].

### 5.2. Persistent Foramen Ovale (PFO)

The prevalence of a PFO in the normal population is estimated to be around 25% and is of potential clinical relevance since the right to left shunt predisposes to the entrance of nitrogen bubbles to the systemic circulation, leading to a DCS [37].

In many patients with CHD, the presence of a PFO is already known or is coincidentally observed during routine echocardiography. In general, the risk of DCS per dive is low in recreational divers (0.01–0.03%). In patients with a PFO, this risk is 4.9–12.9 times higher (5,1 per 10,000 dives) than in divers without a PFO and is related to the size of the shunt [34,38,39]. Currently, there is insufficient evidence to recommend screening on a routine basis in recreational divers or to consider preventive closure of a PFO [40]. Patients with a known PFO could either decide not to start diving or be advised only to participate in conservative diving (Box 1).

After PFO-related DCS, there are three options: discontinuation of diving, conservative diving (Box 1) or unrestricted diving after PFO closure [41,42,43]. Although beyond the scope of this review, PFO closure after DCS with a high probability of a causal relation with the PFO can be considered [40]. PFO closure in these patients is safe with a low burden of recurrent episodes of DCS [44,45]. Patients who suffered from DCS should be counselled against diving until the adequate sealing of the PFO has been confirmed [40].

### 5.3. Ventricular Septal Defect (VSD)

Patients after previous VSD repair without residual shunt, no or mild dilatation of the left ventricle and preserved left ventricular function are fit to dive. Symptomatic arrhythmia is present in 13% to up to 33% of patients after VSD repair, although clinically relevant ventricular tachycardia is rare [28,31]. Most patients after VSD closure during childhood have an adequate systolic left ventricular function, although an abnormal LV function can be found in up to 24% during long-term follow-up [28]. Pulmonary hypertension after VSD closure at a young age is rare, although the prevalence of elevated pulmonary artery pressure is increasing with age [28,46]. In case of pulmonary hypertension, abnormal LV function or relevant ventricular arrhythmias, patients should be counselled against diving. Patients with an unrepaired small, restrictive VSD, or with a small residual shunt after repair, without any signs of substantial volume loading, are allowed to dive. Patients with a large, non-restrictive VSD are advised against diving. These patients have a substantial left ventricular volume-loading and hence are at an increased risk of IPE during diving. Furthermore, pulmonary artery pressure is often elevated in these patients. Patients after recent successful VSD closure are advised not to dive up to 6 (percutaneous closure) or 12 months (surgical closure), with a careful evaluation of cardiopulmonary status before starting diving.

### 5.4. Right Ventricular Outflow Tract (RVOT) Obstruction

RVOT obstruction can occur at several levels. Valvular stenosis is most common and is usually isolated. Sub-infundibular stenosis is rare (double chambered right ventricle) and commonly associated with VSD, while infundibular stenosis is typically seen in tetralogy of Fallot (ToF) [47]. Supravalvular stenosis is extremely rare and often seen in patients with a specific syndrome such as Williams’ syndrome or in patients with congenital Rubella syndrome. Mild RVOT obstruction is usually well-tolerated during exercise and is not a contraindication for diving [48]. In patients with moderate or severe RVOT obstruction (mean PG > 20 mmHg or peak PG > 36 mmHg), the additional increase in pulmonary vascular resistance during diving will lead to an increase in pressure loading of the right ventricle; these patients should be advised against diving [7,8].

### 5.5. Left Ventricular Outflow Tract (LVOT) Obstruction

LVOT obstruction can be either subvalvular, valvular or supravalvular. In patients under 50 years of age, bicuspid aortic valve (BAV) is the most common cause of LVOT obstruction, with a reported birth prevalence of around 1% [47,49]. Many patients with BAV develop aortic stenosis or regurgitation, requiring surgical intervention in up to 50% of patients during their lifespan [50]. Patients with moderate or severe LVOT obstruction (mean PG > 20 mmHg or peak PG > 36 mmHg) should be advised against diving [7,8]. In patients with BAV, specific attention is needed for associated lesions such as aortic dilatation and coarctation. Aortic dilatation is present in up to 80% of BAV patients [49].

### 5.6. Valvular Regurgitation 

Valvular regurgitation is a volume-loading condition for the left or right ventricle. In general, mild to moderate regurgitation without signs of remodeling is well-tolerated during exercise. In general, patients with no or mild to moderate regurgitation of the atrioventricular, aortic, or pulmonary valve, with no or mild dilatation of the left or right ventricle are allowed to dive [7,8]. Patients with severe valvular regurgitation are advised against diving.

### 5.7. Mitral Valve Stenosis

Congenital mitral valve stenosis is rare and consists of a spectrum of defects that will result in obstruction of the left ventricular inflow [51]. The fixed mitral valve area hampers increase in cardiac output and will increase left atrial pressure and eventually pulmonary artery pressure. In addition, there is an increased risk of development of atrial fibrillation and thromboembolic events. Patients with moderate or severe mitral valve stenosis are therefore considered unfit to dive. Only in selected cases with mild mitral valve stenosis with a normal pulmonary artery pressure, diving could be considered. In all cases, we recommend prior cardiopulmonary exercise testing and in selected cases, stress echocardiography to evaluate the mitral valve pressure gradients and pulmonary artery pressure during exercise. 

### 5.8. Aortic Coarctation 

Patients with uncorrected aortic coarctation, or significant re-coarctation after prior coarctation repair, are unfit to dive. The markedly increased blood pressure in the upper body will be increased further by immersion of the body, increasing arterial blood pressure. This is further potentiated by the vasoconstrictive effects of cold water during diving. This uncontrolled hypertension is a risk factor for both the development of immersion pulmonary oedema and DCS [52]. 

After successful aortic coarctation repair, numerous patients remain hypertensive, with the associated long-term detrimental cardiac effects, such as diastolic dysfunction and left ventricular hypertrophy [53]. These factors should be considered when evaluating a coarctation patient. Only patients with successfully repaired aortic coarctation with an adequately (medically controlled) blood pressure, and in the absence of a hypertensive blood pressure response during exercise testing, are allowed to dive. 

Specific attention should be paid to patients who underwent surgical correction by lateral thoracotomy, due to the risk of persisting pleural adhesions. In these patients, we recommend pulmonary evaluation including lung function tests and CT thorax, to rule out any pathology of the lung parenchyma or pleural adhesions [20]. In patients with abnormal test results, consultation of a pulmonologist is advised. 

### 5.9. Aortic Dilatation 

Most cases of thoracic aortic dilatation are caused by degenerative atherosclerotic processes, although, especially in younger patients, many forms of syndromic and familial aorta pathology are increasingly being recognized [54]. Because of the increase in blood pressure during diving and heavy diving equipment, diving is not recommended in patients with a moderate (aorta size ≥ 45 mm, z-score > 4) or severe aortic dilatation. Patients with a syndromic aorta pathology such as Marfan syndrome, Loeys–Dietz syndrome or vascular Ehlers Danlos have an increased risk of dissection and are advised not to dive, regardless of aortic dimensions [55] In addition, Marfan syndrome patients have an additional increased risk of spontaneous pneumothorax and the development of bronchiectasis or pulmonary bullae, which make these patients unfit to dive [56].

### 5.10. Tetralogy of Fallot (ToF)

In the rare patient with an un-operated ToF, the obligate right to left shunting with hypoxemia, poor exercise capacity, increased right ventricular pressure and impaired right ventricular function renders them unfit to dive. 

There is a wide spectrum of sequalae in patients after surgical correction of ToF. Especially during adulthood, many patients develop arrhythmias, most often supraventricular, and/or have an impaired exercise capacity, although right ventricular function is preserved or only slightly impaired in most patients [29]. Significant pulmonary regurgitation is common, and many patients will undergo one or more surgical or percutaneous pulmonary valve implantations during their lifespan. In selected cases, operated ToF patients with mild to moderate pulmonary regurgitation, no or mild right ventricular dilatation, preserved right and left ventricular function and adequate exercise capacity, can be considered fit to dive. In patients with pulmonary stenosis, the recommendations discussed in the section covering RVOT obstruction will apply. Specific attention should be paid to aortic dilatation because aortic aneurysms can be found in ToF patients, although significant progression is rare [57]. Patients with moderate or severe aortic dilatation (Table 2) are advised not to dive. A residual VSD, especially in the presence of pulmonary stenosis, with subsequently elevated right ventricular pressure should be ruled out to prevent right to left shunting. Finally, patients should be free of incapacitating arrhythmias.

### 5.11. Ebstein’s Anomaly

In general, patients with Ebstein’s anomaly are advised not to dive. In these patients, there is a varying degree of tricuspid valve regurgitation and atrialization of the right ventricle with impaired right ventricular function. In addition, patients are prone to developing arrhythmias, the majority based on accessory pathways [58]. An intra-atrial connection is present in 80% to 94% of all patients, and many have an impaired exercise capacity [59,60]. These considerations disqualify most patients with Ebstein’s anomaly from diving. Only in selected cases (forme fruste) without an intra-atrial connection, no arrhythmias, no more than mild to moderate tricuspid regurgitation and a normal right ventricular volume and function, could diving be considered. 

### 5.12. Fontan Circulation 

All patients with Fontan circulation should be counselled against diving, since, in general, they have a clearly impaired exercise capacity, are prone to the development of poorly tolerated supraventricular arrhythmia and have a high occurrence of right to left shunting, either at atrial level or by the development of veno–venous collaterals. In addition, many patients develop systemic AV valve regurgitation or have a diminished systemic ventricular function [61,62,63]. Because heart rate is essential for increasing cardiac output in Fontan patients, the bradycardia induced by the diving reflex will be poorly tolerated and limits their ability to adequately increase cardiac output in an aqueous environment. These considerations make people after Fontan palliation unsuitable for diving. 

### 5.13. Transposition of the Great Arteries (TGA) 

Patients with TGA after atrial switch procedure (Mustard or Senning) have a high risk for developing systemic right ventricle dysfunction, development of heart failure and are prone to atrial arrhythmias [64]. In some patients, significant obstruction of the left ventricular outflow tract (sub-pulmonary ventricle) is present. Furthermore, baffle leaks are common and are a potential risk for right to left shunting. In addition, in many patients, exercise capacity is diminished [64,65]. Patients with congenital-corrected transposition of the great arteries (ccTGA) also show a severely diminished aerobic exercise capacity and frequently present with AV block, necessitating the implantation of an endovascular pacemaker [66]. These considerations make patients with a systemic right ventricle unfit for diving. 

Most patients with TGA after arterial switch have an adequate exercise capacity and are fit-to-dive [33]. Specific attention should be paid to rule out supravalvular pulmonary stenosis, although only 6% of patients have a more than mild supravalvular pulmonary stenosis [67].

### 5.14. Cyanotic CHD

Adults with cyanotic CHD are a heterogeneous group and can be divided into either patients with shunts with normal or restricted pulmonary blood flow, patients with pulmonary vascular disease secondary to a non-restrictive shunt or patients with aortopulmonary connections [68]. Currently, Eisenmenger syndrome is the most severe form with irreversible pulmonary hypertension. Patients with cyanotic heart disease are unfit to dive. In general, patients have a poor exercise capacity and the right to left shunt make these patients prone to the development of DCS. 

### 5.15. Left and Right Ventricular Dysfunction

Patients with an impaired left or right ventricular systolic function are at increased risk of deterioration of a previously well-tolerated condition by the combined increase in preload and afterload that will occur during diving [69]. Because of the already impaired exercise capacity in ACHD patients, it is questionable whether patients with impaired ventricular function can fulfil cardiopulmonary demands in case of a diving emergency [33]. Patients with a more than mildly impaired left ventricular function (left ventricular ejection fraction < 45% or any impaired systemic right ventricular function) should therefore be counselled against diving [8,12,24]. Patients with mild right ventricular dilatation (basal diameter < 42 mm) with preserved systolic function (TAPSE > 17 mm, FAC > 35%) are considered fit to dive.

### 5.16. Arrhythmia and Conduction Disturbances 

Arrhythmia is common in patients with congenital heart disease with atrial arrhythmias complicating 15% of all cases [70,71]. Ventricular tachycardia is less prevalent, and is more common in the more recently operated patients [72]. Patients who develop supraventricular arrhythmias have an increased risk of adverse events with a 50% increase in mortality risk and more than double the risk of stroke or heart failure, warranting thorough examination and a conservative approach in candidate-divers [71]. Several risk factors such as the complexity of the defect (double outlet right ventricle, atrioventricular septal defect, transposition of the great arteries and ToF) but also other factors, such as increasing age, male gender, heart failure and obstructive sleep apnea, have been identified as independent risk factors for arrhythmias [73].

Ventricular tachycardia is incompatible with diving [17,23]. Patients with an ICD for primary or secondary prevention by convention will have underlying diseases that make them unfit to dive. Non-incapacitating premature ventricular complexes with low burden and no disqualifying underlying structural heart disease are no reason to advise against diving. 

Patients with supraventricular arrhythmias should be free from episodes for at least 6 to 12 months before initiating diving. If there have been prior episodes of incapacitating arrhythmias, candidate-divers should be counselled against diving. Patients with permanent atrial fibrillation with adequate rate control and preserved left ventricular function could be allowed to dive, although restrictions might apply. If there is an indication for anticoagulation, the usual precautions and considerations should be considered [74]. In patients with a pre-excitation ECG pattern, an EP study is indicated to rule out any malignant behavior of the accessory pathway [23]. In case of malignant behavior and successful ablation, there is negative diving advice for at least 3 and up to 6 months. During this period, the candidate-diver must be without any recurrent complaints or episodes.

Patients with a first-degree AV block and second-degree AV block-type Wenckebach are allowed to dive. Patients with high-grade AV block, second-degree AV block type Mobitz 2 and total AV block should not dive.

A pacemaker is by itself compatible with diving if the patient is not pacemaker-dependent and the pacemaker has been approved for use under hyperbaric circumstances [75]. 

### 5.17. Prosthetic Valves

In all patients with prosthetic valves, the condition for which the valve was implanted should not be incompatible with diving. It is essential to rule out any complications after surgery and the valve should function satisfactorily for at least 12 months prior to evaluating the subject’s fitness to dive. 

Patients with a well-functioning biological heart valve prosthesis, homograft, or xenografts without any other substantial cardiac abnormalities and preserved left and right ventricular function, are fit to dive. An annual check-up is indicated to rule out any valvular degeneration. 

In general, a mechanical heart valve is compatible with diving, however careful evaluation of additional cardiac pathology is warranted and specific precautions and considerations regarding diving with anticoagulants should be considered (Box 2) [74].

Box 2 **Diving with anticoagulation.**

- *No INR out of range in past 3 months*
- *No combination of two anticoagulatory drugs*
- *No simultaneous use of NSAIDs*
- *No major bleeding in the last year*
- *No additional factors increasing risk of*
- *Pulmonary barotrauma (e.g., asthma, smoking)*
- *No hazardous diving locations*
- *Conservative diving profile*
  ○ *No decompression dives*
  ○ *No dives > 20 m*
  ○ *No > 2 dives per day (>4 h interval)*
  ○ *No dives without possibility direct ascent (no cave/wreck diving)*

After implantation of a mechanical heart valve, patients are advised not to dive in the first year post-operatively as the risk of acute mechanical valve dysfunction and mechanical prosthetic valve thrombosis is the highest in the first period post-operatively [76]. In addition, patients should be completely recovered from the thoracotomy. Patients should only (re)start diving after complete evaluation after this period.

### 5.18. Pulmonary Hypertension

All patients with pulmonary hypertension should be counselled against diving [7,8,27]. In patients with pulmonary hypertension there is an increased risk of right to left shunting (both intracardiac as intrapulmonary) which increases the risk of the development of DCS [77]. In addition, during diving, pulmonary arterial pressure will increase caused by the translocation of blood to the central circulation induced by the increase in hydrostatic pressure, which is a hemodynamic challenge for the right ventricle. Secondly, the pulmonary artery pressure may rise further during after the dive because of the formation of circulating nitrogen bubbles. Recently, in healthy persons, an increase in systolic pulmonary pressure of 18% was observed after recreational scuba diving [14]. 

## 6. Cardiac Medication 

The use of (cardiac) medications may impact the ability of divers to adapt to hyperbaric conditions. Recently, Hoenecamp and colleagues systematically reviewed the interaction of hyperbaric conditions and medication [78]. In this section, the most important aspects of cardiovascular medication in diving are discussed. 

### 6.1. ACE Inhibitors and Angiotensin II Inhibitors

ACE inhibitors and angiotensin II inhibitors are well-tolerated during diving. Specific diving-related risks are not expected. A common side effect of ACE inhibitors is the development of a dry cough, which can be problematic during diving. In general, these drugs are considered safe to use during diving [21].

### 6.2. Beta-Blockers

Beta-blockers are not favorable for use in diving. The induction of bradycardia, augmented by the diving reflex, and reduction in myocardial contractility might hamper the required increase in cardiac output. Furthermore, the reduction in contractility and peripheral vasoconstriction increases the risk of immersion pulmonary edema [12,52]. 

Beta-blockers might also have a pulmonary effect by inhibition of bronchial beta-2 receptors in susceptible individuals [21]. All patients on beta-blockers should undergo both pulmonary function and cardiopulmonary exercise testing during their evaluation [21].

### 6.3. Calcium Antagonists

The dihydropyridine calcium channel blockers are more vascular-selective compared to the non-dihydropyridine calcium channel blockers [79]. Divers using dihydropyridine calcium channel blockers should be aware of the development of orthostatic hypotension when leaving the water. Otherwise, there are no other specific diving-related risks [21]. 

In divers using non-dihydropyridine calcium channel blockers, the indication for using these drugs is more important. These drugs are more myocardium-selective and have a more negative dromotropic and chronotropic effect [79]. Caution should be paid to the development of bradycardia induced by the diving reflex. 

### 6.4. Other Anti-Arrhythmic Drugs

Little is known about the effects of a hyperbaric environment on class 1, 3 and 5 anti-arrhythmic drugs. In general, again, the underlying diagnosis for using these drugs and the symptoms during supraventricular tachycardia should be decisive during the evaluation of a candidate-diver. In general, ACHD patients with not-optimally controlled arrhythmia should be counselled against diving. 

### 6.5. Diuretics

Several classes of diuretics are in use, including thiazide diuretics, loop-diuretics and potassium-sparing diuretics. These drugs are usually not prescribed because they potentiate the development of hypovolemia during diving [21]. More importantly, ACHD patients who use diuretics because of heart failure are disqualified from diving. 

### 6.6. Anti-Thrombotic Drugs 

During diving, there is an increased risk for the development of bleeding complications, mainly related to barotrauma, although several cases of spontaneous bleeding have been described [74]. Antithrombotic drugs comprise vitamin K antagonists (acenocoumarin, warfarin, etc.), antiplatelet drugs (COX inhibitors, P2Y12 inhibitors and phosphodiesterase inhibitors) and direct anti-coagulants (DOACs such as apixaban). The indication for the use of anti-thrombotic drugs should not be incompatible with diving. Currently, there is no consensus regarding the risks of anti-thrombotic drugs in diving [74,80]. Patients on vitamin K antagonists should have a stable INR for at least 3 months with the strong recommendation to measure INR prior to the dive by using point-of-care equipment. Specific recommendations apply for minimizing the nitrogen (over) saturation to limit the risk of developing DCS and minimizing the time needed for a safe resurface (Box 2) [74].

## 7. Ethical Considerations 

Scuba diving can be a wonderful experience, but it also may have a fatal outcome. Adequate training and the use of the proper equipment is of the greatest importance to avoid diving fatalities. If an ACHD patient wishes to engage in scuba diving, evaluation should also include the risk for his/her diving-buddy. The risk of a fatal diving accident due to a cardiac event in non-ACHD divers <50 years is low. Although studies are lacking, ACHD patients are at an increased risk of a (fatal) diving accident due to their underlying condition and the cardiovascular effects of diving. This is especially true for patients with ACHD with moderate and severe complexity. During the evaluation process, these potentially fatal risks should be weighed against the personal motivation to engage in diving. 

## 8. Future Directions 

Due to the great diversity in anatomy, surgical history and residual abnormalities, evaluating fitness to dive in ACHD patients is complex and most recommendations are based on expert opinion. Therefore, evaluating fitness to dive in ACHD patients should be centered in expert centers with adequate diagnostic tools and knowledge of both diving physiology and congenital heart disease. We advocate the start of a prospective international registry to gain more knowledge about diving and diving-related complications to establish more evidence-based recommendations in the future. 

## 9. Conclusions 

Although current ESC guidelines on sports clearly advocate a more liberal standpoint regarding sports participation in general, diving is not considered safe in specific conditions. Since more patients with ACHD wish to start diving, there is an unmet need for clear recommendations on how to evaluate these patients and when to declare them fit-to-dive. A thorough evaluation, including at least an ECG, echocardiography and exercise test, is essential and should be performed by physicians with experience in both diving physiology and the unique considerations and residual abnormalities of congenital heart disease. A stepwise approach is advised to consider possible contraindications for diving such as aortic dilatation, cyanosis and severe ventricular dysfunction. There is a strong need for more data to formulate more robust recommendations in supporting the diving evaluation process in ACHD patients. This paper is a first attempt to set the stage.

## 10. Limitations

This review is limited to recreational open-circuit scuba diving using compressed air or nitrox and not intended for professional diving or for diving with advanced gaseous mixtures, such as trimix or with the use of closed-circuit (“rebreather”) systems. 

Since studies evaluating the safety of diving in ACHD patients are lacking, all advice is based on the personal knowledge and experience of the authors in the areas of ACHD, diving medicine and sports cardiology. 

## Figures and Tables

**Table 1:** Assessment of nine parameters at rest [7,8,24,27,28].

Parameter		Definitions	
Ventricular dysfunction	*No dysfunction*: LVEF > 55%, RV TAPSE > 17 mm, S’ > 10 cm/s, FAC > 35%	*Mild dysfunction*: 45% ≥ EF < 55% (or normal sRV function)	*Moderate–severe dysfunction*: EF < 45% or impaired sRV function
Ventricular hypertrophy	*No hypertrophy*: Wall thickness (cm): <1.1 (male) or <1.0 (female)	*Mild hypertrophy*: Wall thickness (cm): 1.1–1.3 (male) or 1.0–1.2 (female)	*Moderate–severe hypertrophy*: Wall thickness (cm) ≥ 1.3 (male) or ≥1.3 (female)
Ventricular pressure overload	*No pressure overload*: No RVOT or LVOT obstruction (PSV < 2.6 m/s), no coarctation	*Mild pressure overload*: 2.6 m/s ≤ PSV < 3 m/s for LVOT and RVOT obstructions and PPS; for CA, peak arm-leg gradient < 20 mmHg	*Moderate–severe pressure overload*: PSV > 3 m/s for LVOT and RVOT obstruction and PPS, CA peak arm-leg gradient ≥ 20 mmHg
Ventricular volume overload	*No volume overload*: Absent or mild to moderate valve regurgitation without LV/RV dilatation	*Mild volume overload*: Mild to moderate valve regurgitation with mild LV/RV dilatation (LVEDD < 61 mm/RVEDD < 42 mm with preserved systolic function)	*Moderate–severe volume overload*: Severe valve regurgitation or moderate–severe LV/RV dilatation (LVEDD > 61 mm/RVEDD > 42 mm)
Pulmonary artery pressure	*Low probability PH*: TVRVc ≤ 2.8 m/s and no additional echocardiographic findings suggestive of PH or invasive mPAP < 20 mmHg		*Intermediate–high probability PH*: TVRVc > 2.8 m/s or additional echocardiographic findings suggestive of PH or invasive mPAP > 20 mmHg
Aorta (non-syndromic)	*No dilatation*: Aorta size ≤ 35 mm, z-score < 3	*Mild dilatation*: Aorta size ≤ 45 mm, z-score ≤ 4	*Moderate–severe dilatation*: Aorta size ≥ 45 mm, z-score >4 Any syndromic aorta syndrome
Arrhythmia	*No arrhythmia*: Absence of arrhythmia or infrequent PVCs (<500/24 h) that do not worsen during exercise	*Mild arrhythmia*: Frequent PVC not worsening during exercise Controlled AF/AFl or other SVT without incapacitating symptoms	*Clinically important arrythmia*: Any ventricular arrhythmia Any previously incapacitating SVT Pre-excitation pattern without EP study
Arterial oxygen saturation at rest/during exercise	*Normal*: SaO_2_ > 95% in rest or during exercise		*Abnormal*: SaO_2_ < 95% in rest or during exercise
Shunts	*No shunt*: No residual ASD or VSD after closure	*Shunt*: Small, restrictive VSD without LV dilatation PFO	*Shunt*: ASD with R—L shunt VSD with LV dilatation

Abbreviations: LVEF: left ventricular ejection fraction; RV: right ventricle; TAPSE: tricuspid annular plane systolic excursion; FAC: fractional area change; EF: ejection fraction; sRV: systemic right ventricle; LVOT: left ventricular outflow tract; RVOT: right ventricular outflow tract; PPS: peripheral pulmonary stenosis; CA: aortic coarctation; LVEDD: left ventricular end-diastolic diameter; RVEDD: right ventricular end-diastolic diameter; TVRVc: tricuspid valve regurgitation velocity; PH: pulmonary hypertension; mPAP: mean pulmonary artery pressure; PVC: premature ventricular complex; AF: atrial fibrillation; AFl: atrial flutter; SVT: supraventricular tachycardia; EP: electrophysiology; ASD: atrial septal defect; VSD: ventricular septal defect; PFO: persistent foramen ovale.

**Table 2:** CHD diagnoses incompatible with SCUBA diving.

CHD Diagnosis	Feature Relevant to Scuba Diving
Unrepaired Atrial septal defect	R–L shunting, volume overload
Moderate and severe RVOT and LVOT obstruction	Pressure overload, subendocardial ischemia
Severe valvular regurgitation	Volume overload, ventricular dysfunction
Moderate or severe mitral valve stenosis	Impaired cardiac output, pulmonary hypertension, thrombosis
Ebstein anomaly	TR regurgitation, RV dysfunction, ASD, accessory pathway
Unrepaired ToF	R–L shunting, RVOT obstruction, RV dysfunction Pulmonary regurgitation, RV dysfunction, arrhythmia
TGA atrial switch (Mustard/Senning)	sRV dysfunction, arrhythmia, baffle leak
ccTGA	sRV dysfunction, AV conduction disorders
Fontan circulation and cyanotic heart disease	Impaired cardiac output, R–L shunt, arrhythmia, increase pulmonary artery pressure
Unrepaired aortic coarctation or significant re-coarctation	Arterial hypertension
Marfan syndrome or other syndromal aortopathy	Aorta dilatation, increased risk pneumothorax
Non syndromal aortic dilatation With moderate aorta dilatation (aorta ≥ 45 mm, z-score > 4)	Aorta dilatation

Abbreviations: RVOT: right ventricular outflow tract; LVOT: left ventricular outflow tract; TR: tricuspid valve regurgitation; RV: right ventricle; ASD: atrial septal defect; ToF: tetralogy of Fallot; TGA: transposition of the great arteries; sRV: systemic right ventricle; ccTGA: congenitally corrected transposition of the great arteries; AV: atrioventricular.

## Data Availability

Not applicable.
